# Transcriptomic analysis of chloride tolerance in *Leptospirillum ferriphilum* DSM 14647 adapted to NaCl

**DOI:** 10.1371/journal.pone.0267316

**Published:** 2022-04-29

**Authors:** Javier Rivera-Araya, Thomas Heine, Renato Chávez, Michael Schlömann, Gloria Levicán

**Affiliations:** 1 Biology Department, Faculty of Chemistry and Biology, University of Santiago of Chile (USACH), Santiago, Chile; 2 Environmental Microbiology, Institute of Biosciences, TU Bergakademie Freiberg, Freiberg, Germany; Western Michigan University, UNITED STATES

## Abstract

Chloride ions are toxic for most acidophilic microorganisms. In this study, the chloride tolerance mechanisms in the acidophilic iron-oxidizing bacterium *Leptospirillum ferriphilum* DSM 14647 adapted to 180 mM NaCl were investigated by a transcriptomic approach. Results showed that 99 genes were differentially expressed in the adapted versus the non-adapted cultures, of which 69 and 30 were significantly up-regulated or down-regulated, respectively. Genes that were up-regulated include carbonic anhydrase, cytochrome *c* oxidase (*ccoN*) and sulfide:quinone reductase (*sqr*), likely involved in intracellular pH regulation. Towards the same end, the cation/proton antiporter CzcA (*czcA*) was down-regulated. Adapted cells showed a higher oxygen consumption rate (2.2 x 10^−9^ ppm O_2_ s^-1^cell^-1^) than non-adapted cells (1.2 x 10^−9^ ppm O_2_ s^-1^cell^-1^). Genes coding for the antioxidants flavohemoprotein and cytochrome *c* peroxidase were also up-regulated. Measurements of the intracellular reactive oxygen species (ROS) level revealed that adapted cells had a lower level than non-adapted cells, suggesting that detoxification of ROS could be an important strategy to withstand NaCl. In addition, data analysis revealed the up-regulation of genes for Fe-S cluster biosynthesis (*iscR*), metal reduction (*merA*) and activation of a cellular response mediated by diffusible signal factors (DSFs) and the second messenger c-di-GMP. Several genes related to the synthesis of lipopolysaccharide and peptidoglycan were consistently down-regulated. Unexpectedly, the genes *ectB*, *ectC* and *ectD* involved in the biosynthesis of the compatible solutes (hydroxy)ectoine were also down-regulated. In line with these findings, although hydroxyectoine reached 20 nmol mg^-1^ of wet biomass in non-adapted cells, it was not detected in *L*. *ferriphilum* adapted to NaCl, suggesting that this canonical osmotic stress response was dispensable for salt adaptation. Differentially expressed transcripts and experimental validations suggest that adaptation to chloride in acidophilic microorganisms involves a multifactorial response that is different from the response in other bacteria studied.

## Introduction

*Leptospirillum ferriphilum* is a Gram-negative, and obligately aerobic iron-oxidizing chemoautotroph able to thrive in a pH range from 1.3 to 2.0 [1]. This bacterium belongs to the bioleaching microbial communities involved in solubilization of metals from sulfide ores [2,3].

*L*. *ferriphilum*, like most acidophilic microorganisms, shows extreme sensitivity to chloride and other anions (with the notable exception of sulfate) [4]. Acidophiles possess positive membrane potentials which facilitates the influx of permeable anions into cells [5]. The mass entry of chloride and other anions favor the influx of protons causing the collapse of positive internal potential, and therefore the disruption of the proton motive force, as well as acidification of the cytoplasm and a general detrimental effect in the cell [4,6,7]. Nevertheless, the molecular basis of the response to chloride in *L*. *ferriphilum* and other acidophilic microorganisms are poorly understood.

The mechanisms operating in acidophiles in response to chloride have been investigated just during recent years. In *L*. *ferriphilum*, the osmotic stress induced by sodium chloride leads to the up-regulation of genes encoding the potassium transporter Kdp, and for the biosynthesis or uptake of compatible solutes such as (hydroxy)ectoine and trehalose [8–11]. In members of the genus *Acidithiobacillus*, like *At*. *ferrooxidans* and *At*. *caldus*, the use of proline and betaine as osmoprotectants has been reported [12,13], whilst the moderately halotolerant *Acidihalobacter prosperus* has a response based on the synthesis and uptake of ectoine [14]. In addition, *A*. *prosperus* also seems to have developed a more specific adaptive response that involves changes in the amino acid composition of rusticyanin to protect the copper ion present in the active site of this protein [14].

To respond to cytoplasm acidification induced by chloride exposure, acidophiles synthesize a greater number and diversity of cation/H^+^ antiporters, proteins that modify the cell membrane, and proteins of the electron-transport chain. These changes result in the presumed export of protons, at the expense of increasing the respiratory rate [1,4,14,15]. Recently, Rivera-Araya et al. [4] described that exposure of *L*. *ferriphilum* to chloride led to a significant increase in intracellular reactive oxygen species (ROS). It is believed that ROS enhancement is produced by the increment in respiratory activity and by disruption of metallic centers of proteins due to osmotic imbalance. In addition, Fe^2+^ and other cations can trigger Fenton chemistry and induce the generation of hydroxyl radicals [16]. In agreement with these observations, the activation of antioxidant mechanisms seems to play an important complementary role in the response to chloride. The exposure of *L*. *ferriphilum* to 50–150 mM NaCl has been shown to up-regulate the activity of thioredoxin and cytochrome *c* peroxidase [4]. Similarly, in other microorganisms, like *At*. *caldus* and *Acidimicrobium ferrooxidans*, the up-regulation of antioxidative proteins in response to NaCl has also been reported [7,13].

Therefore, based on the evidence from the individual studies described above, it is possible to state that in *L*. *ferriphilum* and other acidophilic microorganisms, the exposure to chloride triggers a response that involves the participation of different mechanisms to withstand osmotic, acid and oxidative stress. However, it is envisioned that a chloride challenge activates a global and complex physiological response that has yet to be well deciphered. In the present study, we report on transcriptomic analyses conducted in *L*. *ferriphilum* DSM 14647 adapted and exposed to 180 mM NaCl. This study also included the measurements of specific parameters such as oxygen consumption rate, intracellular pH, and ROS and (hydroxy)ectoine content.

## Materials and methods

### Bacterial strains and growth conditions

*L*. *ferriphilum* DSM 14647 [17] used in this study was provided by Leibniz Institute DSMZ. The bacterial cells were cultured in DSMZ 882 medium (pH 1.8) supplemented with 72 mM ferrous sulfate (FeSO_4*_7H_2_O). Bacterial growth was carried out in Erlenmeyer flasks at 180 rpm and 37°C.

### Adaptation of *L*. *ferriphilum* DSM 14647 to 180 mM NaCl

The adaptation of *L*. *ferriphilum* DSM 14647 was performed in growth medium (see above) with increasing NaCl concentrations (50-100-120-150-180 mM) and supplementation with 1 mM ectoine as a compatible solute (Sigma-Aldrich). The adaptation was performed sequentially and with 3 passages per salt concentration. Cultures were maintained until the late exponential phase and used to inoculate fresh NaCl and ectoine-containing medium (10% v/v) and generate a new culture. After the 180 mM NaCl-adapted culture had been obtained, the compatible solute was gradually (1–0.5–0 mM) removed from the medium. Adapted cells were constantly grown in the presence of 180 mM NaCl.

### Growth curve determination

The experiment was carried out in 250 mL Erlenmeyer flasks. Each flask contained 100 mL DSMZ 882 medium with 0 or 180 mM NaCl for non-adapted and adapted cells, respectively. Samples were taken periodically for determination of cell growth, which was measured by direct microscopic counting using a modified Neubauer chamber. The initial cell density was 1 x 10^6^ cells mL^-1^.

### Measurement of minimum inhibitory concentrations (MIC) of NaCl

This assay was carried out on planktonic cells according to Rivera-Araya et al. [11] with some modifications. Briefly, to determine the MIC of NaCl, non-adapted and adapted cells of *L*. *ferriphilum* were cultured in DSMZ 882 medium at pH 1.4, 1.8, 2.4 or 3.0 in the presence of different NaCl concentrations, ranging from 0 to 600 mM. The experiments were performed in triplicate in 6-well plates, each well containing 5 mL of the medium. Bacteria were inoculated to a concentration of 1 x 10^6^ cells mL^-1^ and later incubated at 37°C for 72–86 h, until the control sample (without salt) reached the stationary phase. The MIC value corresponds to the minimal NaCl concentration where no bacterial growth was observed.

### mRNA isolation and transcriptomic analysis

#### mRNA isolation

Cells from control (non-adapted, non-exposed to NaCl), and adapted in 180 mM NaCl conditions were grown until the late exponential phase. Cells were harvested by centrifugation at 8,000 x *g* for 15 min (at 4°C) and washed once with cold 10 mM H_2_SO_4_ and twice with 10 mM sodium citrate pH 7.0. Total RNA was isolated using the RNeasy Mini Kit (Qiagen). DNA was removed by DNase I treatment (New England, Biolabs) according to the manufacturer’s instructions.

#### cDNA library preparation and Illumina sequencing

The quality and integrity of the total RNA were evaluated using an Agilent Bioanalyzer 2100 and an RNA 600 Nano Kit (Agilent Technologies). Three RNA preparations of high quality (RNA integrity number above 7) were pooled together and submitted for transcriptome analysis as previously described [18,19]. Before library preparation, ribosomal RNA was depleted using the MICROBExpress kit (Thermo Fisher). Then, a TruSeq stranded mRNA library prep kit (Illumina) was used to generate cDNA libraries for whole transcriptome analysis. The resulting libraries were sequenced on an Illumina MiSeq system with v3 chemistry and 2 x 75-nucleotide read lengths (paired end).

#### Differential expression analysis

Raw reads from RNA sequencing of non-adapted and adapted cells were processed to remove adaptors, and filtered to obtain reads with a quality higher than Q20, by using the CLC Genomics Workbench software. Then, the filtered reads were aligned onto the reference genome of *L*. *ferriphilum* DSM 14647 [NCBI accession number: PGK00000000] by CLC Genomics Workbench software. Transcriptomic data was submitted to European Bioinformatic Institute database (ArrayExpress Accession: E-MTAB-11136)

Raw counts for each ORFs features were subjected to differential analysis with statistical R software, using the DESeq2 package [20]. A gene was considered differentially expressed with a p-value < 0.05. The assignment of genes to a functional category was carried using the Go Feat Tool and the public Gene Ontology (GO) database [21].

### Oxygen consumption

The oxygen consumption rate was determined by means of optodes (Fibox 3, PreSens-Precision Sensing GmbH, Regensburg, Germany) [22]. In short, fresh iron-grown 100 mL-cultures of *L*. *ferriphilum* DSM 14647 were harvested by centrifugation at 8,000 x *g* for 15 min, the supernatant was removed, and the pelleted cells were resuspended in 0.1 mL the remaining growth medium, before being added to a 3-mL cuvette containing 2.6 mL of DSM 882 culture medium pH 1.8 with 0 or 180 mM NaCl for non-adapted and adapted cell cultures, respectively. Afterwards, 0.15 mL of ferrous iron solution were added to the cuvettes (final concentration 72 mM), and the suspension mixed cautiously. The cuvette was then carefully closed with a glass lid. An oxygen-sensing optode spot had previously been embedded inside the measuring cuvette. Fibre-optics located outside the cuvette on the opposite side of the oxygen sensor spot were connected with a 4-channel fiber–optics oxygen meter (Firesting O_2_), also equipped with a receptacle for a temperature sensor. The optode signal was evaluated using the software Pyro Oxygen Logger. Due to the strong temperature dependence of fluorescence, measurements were performed in a thermostatic cabinet (UVP Hybridizer HB-1000) at 37°C. Optode measurements were performed in triplicate using biological replicates.

### Analysis of intracellular (hydroxy)ectoine content

The compatible solutes ectoine and hydroxyectoine were quantified by HPLC analysis, using an Ultimate 3000–2015 HPLC (Thermo Scientific) system with a 250 mm × 4.6 mm Hypurity Aquastar C-18 column with particle size of 5 μm (Thermo Scientific), as described previously [4]. Chromatography was performed with a gradient of two solutions as mobile phase—eluent A (0.8 mM KH_2_PO_4_, 6.0 mM Na_2_HPO_4_, pH 7.6) and eluent B (acetonitrile)—at a flow rate of 1.0 mL min^-1^ at 25°C. The presence of compatible solutes was monitored at 215 nm by a UV/VIS detector. The retention times of ectoine and hydroxyectoine were determined using commercially available compounds (purity ≥ 95%, Sigma-Aldrich) as standards. Intracellular ectoine and hydroxyectoine content was calculated as ng mg^-1^ of wet biomass, using a calibration curve.

### Determination of ROS levels

The intracellular level of total ROS was measured in non-adapted and adapted cultures using the fluorescent probe 2’,7’-dichlorodihydrofluorescein diacetate (H_2_DCFDA) according to Ferrer et al. [23]. Fluorescent emission values were normalized to the total protein concentration. Protein quantification was performed by the colorimetric Bradford assay [24]. Since ROS determination included a last incubation step with the fluorescent probe under neutral pH conditions, the viability of the cell cultures was tested. For this purpose, a control was performed by incubating cells in 100 mM potassium phosphate buffer pH 7.4 without the probe and then re-inoculating them into fresh medium as described [4].

### Statistical analysis

Statistical analysis was performed using the one-way ANOVA test followed by Tukey’s test in GraphPad Prism 5. The differences were considered to be significant at p < 0.05.

## Results and discussion

### Characterization of growth and NaCl-tolerance of *L*. *ferriphilum* DSM 14647 adapted to 180 mM NaCl

**Fig 1:**
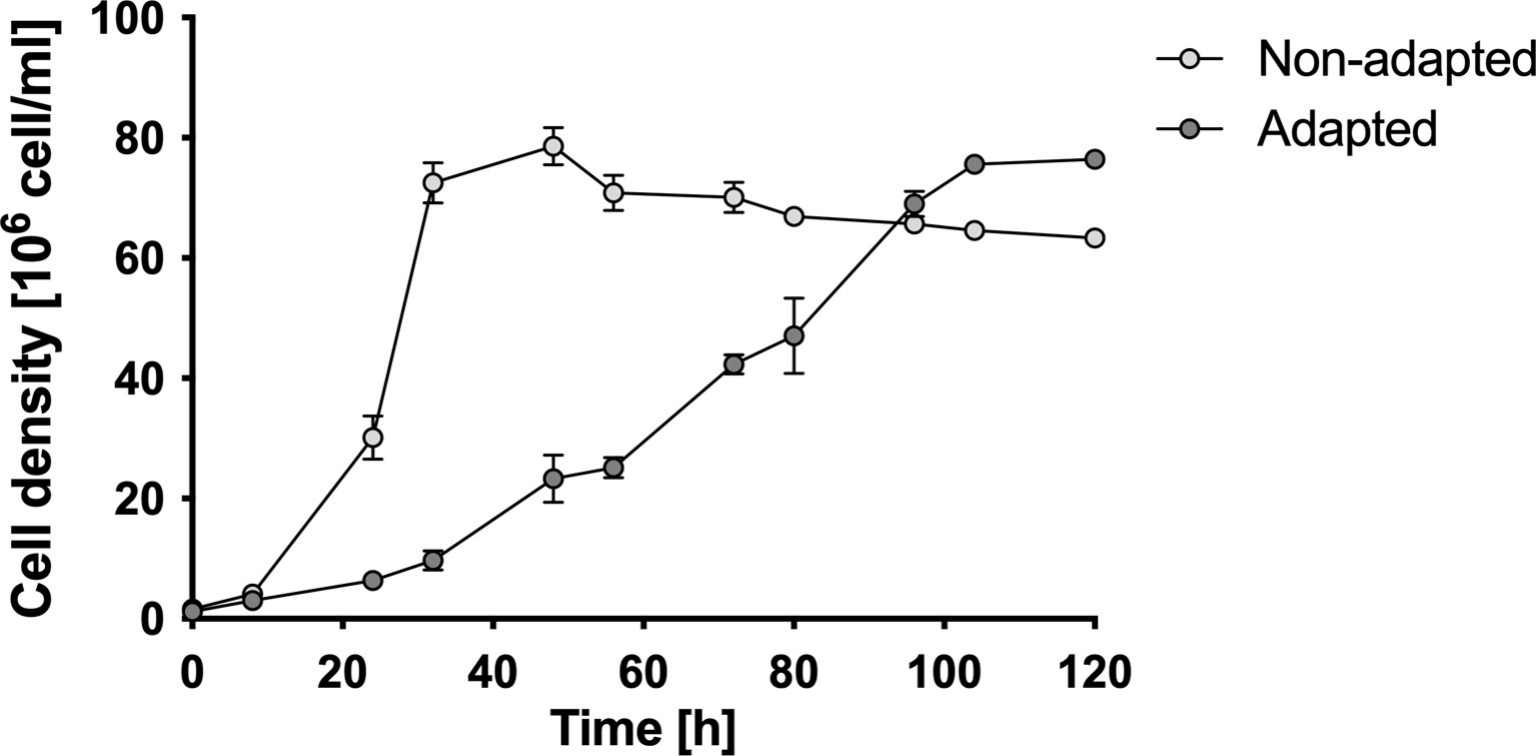
Growth of *L*. *ferriphilum* DSM 14647. Curves represent the growth of adapted and exposed to 180 mM NaCl (grey circles), and non-adapted and non-exposed (white circles) cell cultures. Data represent the average of 3 independent experiments. Error bars represent standard deviation. Initial cell density, 1x10^6^ cells mL^-1^.

The adaptation of *L*. *ferriphilum* to 180 mM NaCl led eventually to a culture with the same cell density (8 x 10^7^ cells mL^-1^) as the non-adapted cell culture (**Fig 1**). However, salt approximately tripled the time of cellular duplication (t_d_) from 6 to 17 h. A retarding effect on growth rate and iron oxidation has been observed in different studies of NaCl-susceptible acidophilic microorganisms, including *L*. *ferriphilum* and other species (*At*. *ferrooxidans* and *S*. *thermosulfidooxidans*) [14,25].

It is important to highlight that although the addition of ectoine favored the sequential acclimation of *L*. *ferriphilum* to 180 mM NaCl (data not shown), the adapted cell culture could grow steadily without ectoine supplementation, indicating that cells were physiologically adapted to face this stress condition.

It has been widely reported that decreasing the external pH contributes significantly to the toxicity of chloride in this species and in other acidophilic bacteria [4,15]. In agreement with this, Fe^2+^ oxidation in the presence of NaCl is highly influenced by the pH of the growth medium [5]. Thus, in order to evaluate the tolerance of NaCl-adapted cells, we determined the MIC of adapted and non-adapted cell cultures exposed to a range of pH values. As shown in **Table 1**, the MIC of the adapted culture was higher than that of the non-adapted culture. In addition, the MIC significantly increased as the pH of the medium increased within the range of 1.4–3.0. However, it was also observed that at a higher pH of the medium, the difference of the MIC between adapted and non-adapted cultures was lower. For example, at pH 1.4 the adapted culture had a MIC value twice (350 mM) that of the non-adapted culture (175 mM), while at pH 3.0, the MIC of the adapted culture was just 25% higher (500 mM) than that of the non-adapted culture (400 mM). The results clearly show that the prior adaptation of *L*. *ferriphilum* conferred a higher tolerance against NaCl, but that this tolerance was more noticeable at a low external pH.

**Table 1:** Minimum inhibitory concentration of NaCl in *L*. *ferriphilum* DSM 14647 adapted to 180 mM NaCl at different external pH (pH_ex_).

pH_ex_	NaCl MIC [mM]	
	*L*. *f*. non-adapted	*L*. *f*. adapted to 180 mM NaCl
1.4	175	350
1.8	225	375
2.4	350	425
3.0	400	500

### Transcriptomic profile of *L*. *ferriphilum* DSM 14647 adapted to 180 mM NaCl

#### Screening of differentially expressed genes (DEG)

The differential expression analysis was performed comparing cultures adapted to 180 mM NaCl versus non-adapted non-exposed cell cultures as described in Materials and Methods. In this analysis, 99 out of 2736 genes showed significant differential expression (p<0.05) of which 69 (2.5%) and 30 (1.1%) were up-regulated and down-regulated, respectively. **Table 2** lists the genes that were up-regulated (excluding 43 ORFs predicted as hypothetical proteins, **S1 Table**) in a range of 4.1- to 91.7–fold change. **Table 3** shows the down-regulated genes (excluding 8 ORFs predicted as hypothetical proteins, **S2 Table**) in a range of -4.3 to -9.3–fold change. Classification of genes by their functionality revealed a number of genes involved in metabolism and energy conservation, the cell envelope, transport and osmoregulation, and stress response and signal transduction, among others.

**Table 2:** Up-regulated genes in *L*. *ferriphilum* DSM 14647 adapted to 180 mM NaCl in relation to non-adapted non-exposed control cells.

Accession number	Gene product	Fold change
**Metabolism and energy conservation**		
KGA94808.1	Carbonic anhydrase (CA)	8.1
WP_036082816.1	Cytochrome *c* oxidase subunit CcoN	4.9
KGA94222.1	Sulfide:quinone reductase (Sqr)	4.6
**Cell envelope**		
WP_014961534.1	Regulator of protease activity HflC	6.6
WP_081938081.1	Fatty acid desaturase	4.9
**Transport and osmoregulation**		
WP_036082891.1	Outer membrane efflux protein TolC	7.4
**Stress response**		
WP_036083168.1	Flavohemoprotein	14.3
WP_052157908.1	Cytochrome *c* peroxidase	10.9
WP_023524701.1	Heat-shock protein Hsp20	8.1
KGA93200.1	Transcriptional Regulator IscR	7.2
WP_036079670.1	FAD-dependent pyridine nucleotide-disulfide oxidoreductase	5.6
KGA93006.1	Radical SAM domain protein	5.2
KGA94361.1	Mercuric reductase, MerA	4.3
**Signal transduction**		
WP_049713715.1	Diguanylate cyclase/phosphodiesterase	11.2
WP_036081469.1	DSF synthase (RpfF)	10.7
WP_161781719.1	Diguanylate cyclase/phosphodiesterase	6.6
WP_036081415.1	Diguanylate cyclase/phosphodiesterase	5.2
**Others**		
KGA93962.1	Transposase	7.7
WP_036081724.1	Phage related integrase	5.4
KGA94115.1	Methyl-accepting chemotaxis protein	5.4
WP_036083266.1	Methyl-accepting chemotaxis protein	5.1
WP_161781749.1	Periplasmic serine protease DO (HtrA)	4.9
WP_036081132.1	Flagellin protein FlaB	4.4
WP_036080943.1	DNA-binding protein HU	4.3
WP_020859441.1	Prokaryotic ubiquitin-like protein Pup	4.2
WP_036082283.1	Shufflon-specific DNA recombinase	4.2

a: Transcriptomic data can be found in EBI database (https://www.ebi.ac.uk/) as indicated in Materials and Methods.

b: Values correspond to the average fold change of 3 biological replicates.

**Table 3:** Down-regulated genes in *L*. *ferriphilum* DSM 14647 adapted to 180 mM NaCl in relation to non-adapted non-exposed control cells.

Accession number^a^	Gene product	Fold change
**Metabolism and cell envelope**		
WP_036081541.1	Glycosyl transferase, group 1 family protein	-5.3
WP_036081614.1	Glutamine-fructose-6-phosphate aminotransferase	-5.7
WP_036081511.1	Glycosyl transferase family 2 protein	-5.3
WP_036081618.1	UDP-glucose dehydrogenase	-6.4
WP_036081521.1	UTP-glucose-1-phosphate uridylyltransferase	-9.0
WP_036081553.1	Undecaprenyl-phosphate galactose phosphotransferase	-4.8
WP_036081550.1	Polysaccharide export protein	-4.9
WP_036081600.1	dTDP-glucose 4,6-dehydratase	-5.0
WP_052157773.1	Glycosyltransferase involved in cell wall biosynthesis	-6.0
WP_036081557.1	Tyrosine-protein kinase EpsD	-6.9
WP_036081546.1	Polysaccharide deacetylase	-8.4
WP_036081519.1	Eight transmembrane protein EpsH	-9.3
**Transport and osmoregulation**		
WP_036080892.1	Outer membrane efflux protein TolC	-5.1
WP_036080895.1	RND efflux transporter	-5.4
WP_036080909.1	RND family efflux transporter MFP subunit	-6.2
WP_036081492.1	ABC transporter ATP-binding protein MdlB	-5.9
WP_020859429.1	Diaminobutyrate-2-oxoglutarate transaminase (EctB)	-6.5
WP_020859430.1	L-ectoine synthase (EctC)	-5.6
WP_020859431.1	Ectoine hydroxylase (EctD)	-8.0
**Stress response**		
WP_036080906.1	Cobalt-zinc-cadmium resistance protein CzcA	-6.8
WP_036080898.1	Two component sigma54 specific transcriptional regulator	-4.3
WP_052157774.1	Sigma-54 dependent transcriptional regulator	-10.2

a: Transcriptomic data can be found in EBI database (https://www.ebi.ac.uk/) as indicated in Materials and Methods.

b: Values correspond to the average fold change average of 3 biological replicates.

#### Metabolism and energy conservation

The adaptation of *L*. *ferriphilum* to 180 mM NaCl resulted in the identification of a number of DEGs related to metabolism and energy conservation. A significant increase in the expression of a carbonic anhydrase (CA, 8.1-fold) was observed in the adapted culture. This metalloenzyme catalyzes the reversible hydration of carbon dioxide to form bicarbonate ions (HCO_3_^-^) and protons in the reaction: CO_2_ + H_2_O ⇔ HCO_3_− + H+ [26]. In autotrophic bacteria that fix CO_2_ through the Calvin-Benson-Bassham cycle, CA is involved in the transport and supply of CO_2_ to Rubisco (D-ribulose 1,5-bisphosphate carboxylase/oxygenase) in the carboxysome [27]. However, since this enzyme produces and uses protons and bicarbonate ions, it also plays a key role in the regulation of pH [28]. In acidophiles, genes encoding CA and the carboxysomal shell proteins have been described in *At*. *ferrooxidans* and *At*. *thiooxidans* [29–31]. Moreover, in *At*. *ferrooxidans*, the expression of the *cbb5* operon that encodes the inorganic carbon transporter SulP and CA is dependent on the CO_2_ concentration regimen [31]. In *L*. *ferriphilum* and other leptospirilli, carbon fixation is performed by the reductive tricarboxylic acid cycle (RTCA) [29] in which, as far as it is known from the literature, CA does not seem to play a role. Thus, the predicted CA of *L*. *ferriphilum* could play a major role by contributing towards neutralizing the acidification of the cytoplasm that is expected to occur in the presence of chloride. In this way, the up-regulation of the CA-encoding gene could represent a direct strategy of cellular pH homeostasis. The contribution of CA to this purpose deserves to be experimentally addressed.

**Fig 2:**
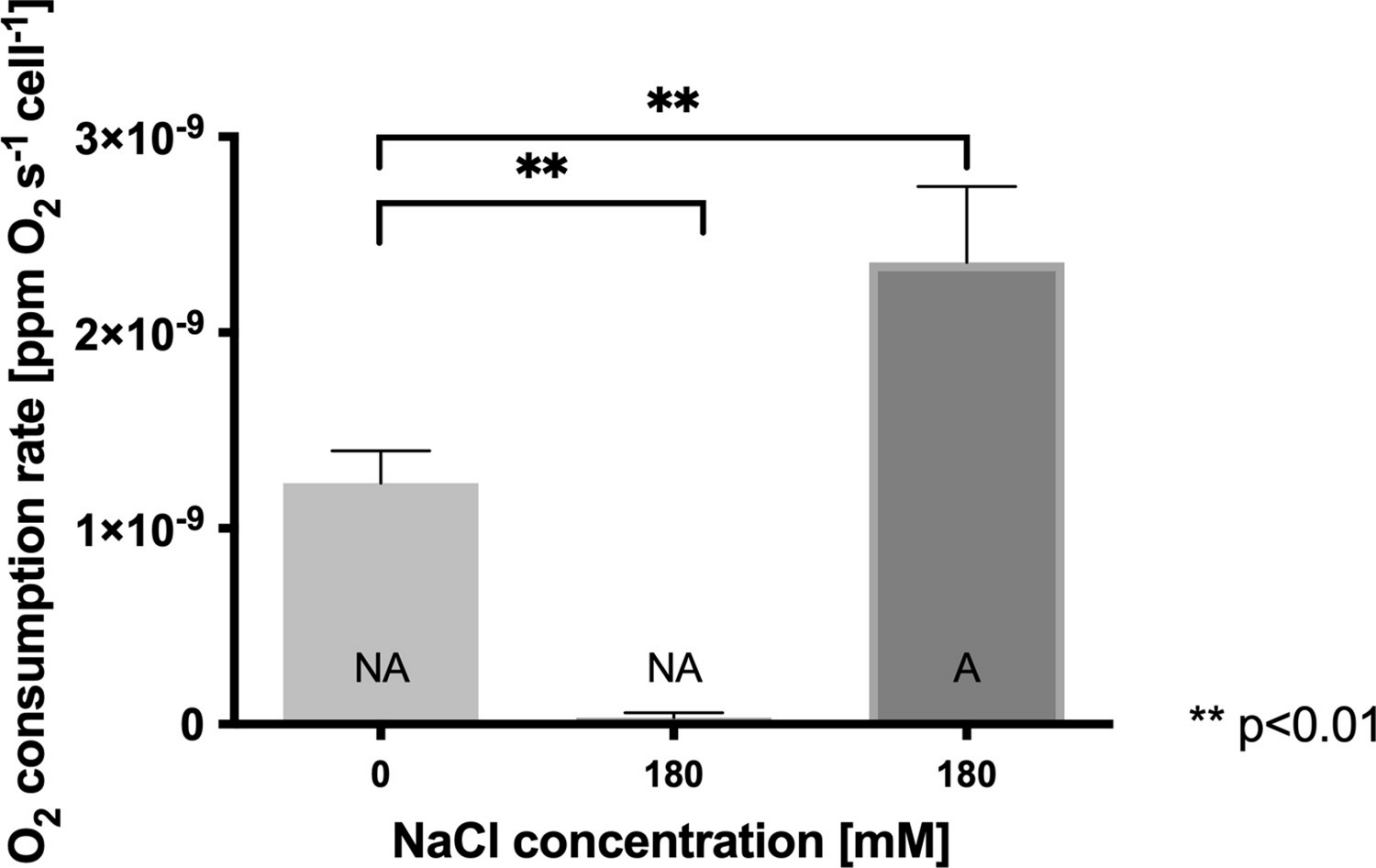
Effect of NaCl adaptation on oxygen consumption rate in *L*. *ferriphilum* DSM 14647. The measurements were carried out in adapted (A) cells exposed to 180 mM NaCl and non-adapted (NA) cells exposed to 0 or 180 mM NaCl. The data represent the average of 3 independent experiments. Error bars represent standard deviation. Statistical analysis was carried out by an ANOVA test.

Genes coding for proteins from electron-transport chains such as cytochrome *c* oxidase CcoN subunit (4.9-fold) and sulfide:quinone reductase Sqr (4.6-fold) were also significantly up-regulated. CcoN is the component of the *cbb*_*3*_-type cytochrome oxidase, a complex enzyme of the respiratory chain which has previously been reported in *Leptospirillum* spp. [32]. CcoN is the catalytic subunit of the enzyme in charge of the four-electron reduction of molecular oxygen to water, a process which is coupled to translocation of protons across the membrane [33]. The Sqr enzyme could play a role in the detoxification of endogenously generated H_2_S, a common product of cysteine metabolism that negatively impacts the redox status of bacterial cells [34,35]. The enzyme obtains electrons from H_2_S oxidation and transfers them to the quinone pool, thus increasing the activity of the electron-transfer chain. The increase of both cytochrome *c* oxidase and Sqr activities should increase the respiratory rate of this bacterium to provide energy (ATP), reducing power (NAD(P)H), and mainly the possibility of extruding protons from the cytoplasm to avoid acidification induced by chloride exposure [4,14]. In order to evaluate whether adapted cells showed a higher respiratory rate, the oxygen consumption of non-adapted and adapted cells of *L*. *ferriphilum* exposed to 180 mM NaCl was measured. As shown in **Fig 2**, non-adapted *L*. *ferriphilum* exposed to 180 mM NaCl was not able to respire. Interestingly, the O_2_ consumption rate in adapted cell cultures treated with 180 mM NaCl was significantly greater than that of non-adapted untreated cells (1.2 x 10^−9^ versus 2.2 x 10^−9^ ppm O_2_ s^-1^cell^-1^; p<0.01). This result supports the idea that up-regulation of electron-transport chain genes contributes towards the increase in the oxygen respiratory activity in adapted cells exposed to NaCl. A similar effect was observed in a proteomic study of *Ac*. *prosperus* in which cytochrome *c*_*1*_, rusticyanin and ATP synthase subunit *b* were over-expressed in the presence of 500 mM NaCl [14], indicating that proton extrusion by respiration may be a widely distributed chloride response mechanism in acidophiles.

#### Cell envelope

One of the up-regulated genes codes for the regulator HflC (6.6-fold) which modulates the FtsH protease and may serve to maintain quality control of some membrane proteins [36,37]. Additionally, a gene coding for a fatty acid desaturase, which belongs to a group of enzymes in charge of double-bond insertion at specified positions of fatty acyl chains, necessary for membrane-lipid fluidity [38], was up-regulated (4.9-fold). In *Synechocystis*, strains overexpressing a desaturase gene were found to be more robust under salt stress conditions [39]. In addition, a correlation between the unsaturation of fatty acids in membrane lipids and tolerance to salt stress in this genus and other bacteria has been reported [39,40]. For *L*. *ferriphilum*, the up-regulation of the fatty acid desaturase gene suggests an increase in the unsaturated/saturated fatty acid ratio. Whether this confers higher fluidity to the membrane in salt stress compared to normal conditions, or it is to compensate a salt-induced decrease in the fluidity and thus ensure a fluid membrane at high salt, remains to be determined.

Several genes involved in carbohydrate metabolism had lower expression in adapted versus non-adapted cells. Among them were genes encoding two glycosyl transferases (-5.3 fold), a UDP-glucose dehydrogenase (-6.4 fold) and a UTP-glucose-1-phosphate uridylyltransferase (-9.0 fold) which are directly related to the synthesis of glycosaminoglycans, critical precursors of peptidoglycans and other cell-surface polymers, such as lipopolysaccharides [41–43]. Another significantly repressed gene under high-salt conditions was glutamine-fructose-6-phosphate aminotransferase/glucosamine-6-phosphate synthase (-5.7 fold), a dimeric enzyme that catalyzes the first step in hexosamin metabolism, converting D-fructose-6-phosphate (Fru6P) and glutamine (Gln) into D-glucosamine-6-phosphate (GlcN6P) and glutamate [44]. The end product of the hexosamine pathway, uridine diphosphate N-acetylglucosamine (UDP-GlcNAc), plays an important role as a precursor of peptidoglycan and glycolipids [45].

Other genes related with the biosynthesis of the cell envelope that were down-regulated in *L*. *ferriphilum* grown at 180 mM NaCl encode undecaprenyl-phosphate galactose phosphotransferase (-4.8 fold) and dTDP-glucose 4,6-dehydratase (-5.0 fold), two enzymes involved in the generation of intermediate nucleotide sugars for O-antigen polysaccharide biosynthesis in the biogenesis of the outer membrane [46,47]. Altogether, these findings suggest that synthesis of cell surface polymers such as peptidoglycan and lipopolysaccharide were diminished as a result of the physiological salt adaptation in *L*. *ferriphilum*. Abiotic stressors jeopardize the integrity of peptidoglycans and other components of the cell envelope by introducing lesions, which must be rapidly repaired to prevent cell lysis [48]. As a consequence, upon osmotic stress induction, cells respond by upregulating the activity of enzymes or genes essential for cell wall synthesis [49]. Thus, based on these antecedents, we envisioned that adapted cells of *L*. *ferriphilum* exposed to 180 mM NaCl did not generate the corresponding response to osmotic stress.

#### Transport and osmoregulation

The adaptation of *L*. *ferriphilum* to NaCl also resulted in the up-regulation of several genes encoding proteins related to cellular transport. These included the gene encoding TolC protein (7.4-fold), a key component of multidrug efflux systems such as AcrAB-TolC, AcrEF-TolC, EmrAB-TolC and MacAB-TolC of the outer membrane, which are important for bacterial survival and oxidative stress responses in acidic environments [50,51].

Conversely, genes encoding several transporters were repressed. In agreement with decreasing the biosynthesis of surface polymers, the expression of a gene encoding a polysaccharide-transport protein implicated in the export of polysaccharides across the outer membrane [52] was significantly lower in salt-adapted cells (-4.9-fold). Genes encoding an outer-membrane efflux protein TolC (different from the one referred to above; -5.1-fold), two genes coding for RND (Resistance-Nodulation-Division) efflux transporters (-5.4 and -6.2-fold, respectively) that form complexes with AcrAB-TolC, and play a role in the active efflux of antimicrobial agents [53], and ABC transporter ATP-binding protein MdlB (-5.9 fold), which is an integral membrane protein named Mdl (Multidrug resistance-like) that actively transports molecules across the lipid membrane against a concentration gradient, were also reduced in expression [54,55].

**Fig 3:**
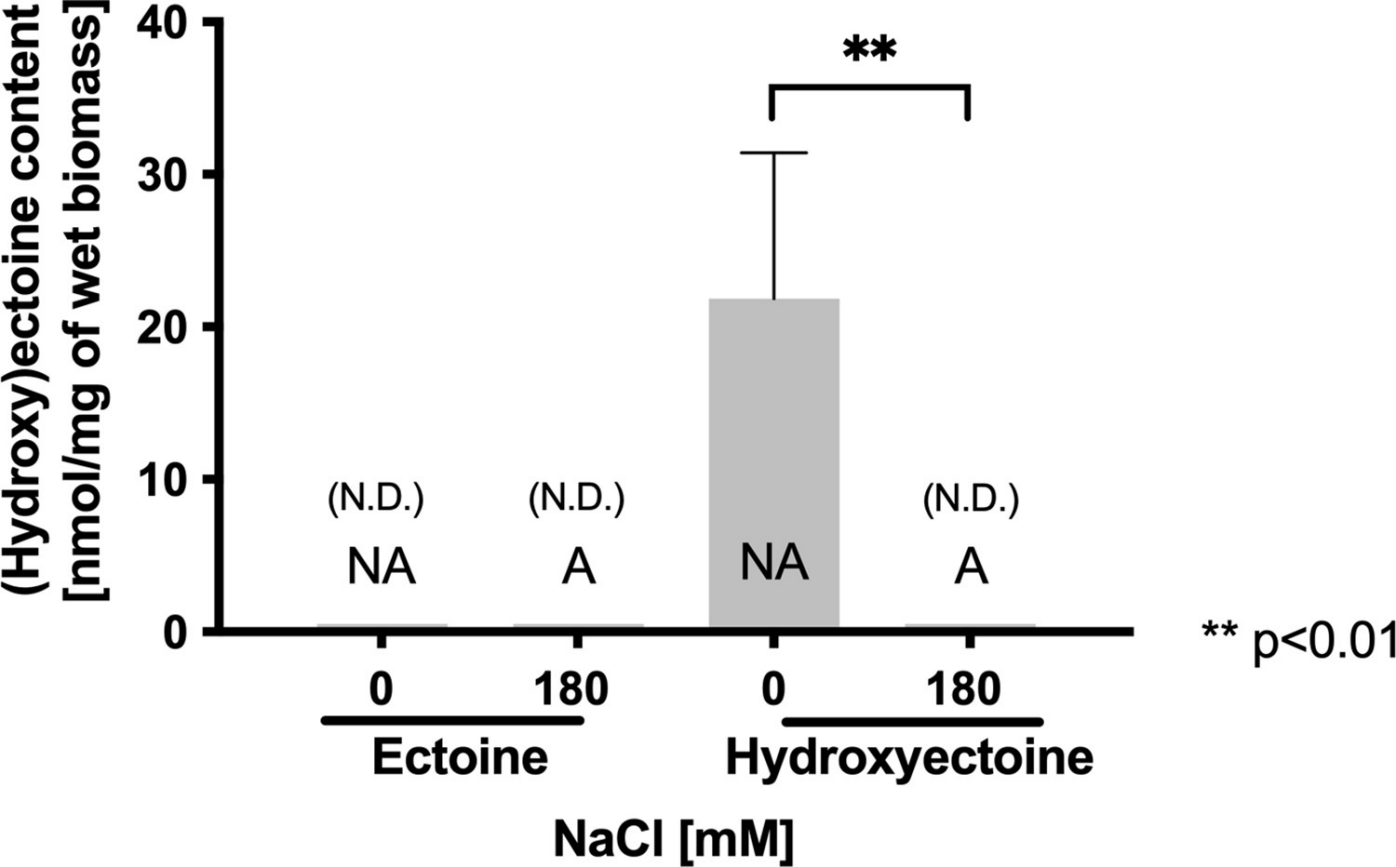
Effect of NaCl adaptation on the content of compatible solutes in *L*. *ferriphilum* DSM 14647. Ectoine and hydroxyectoine content was measured in adapted cells exposed to 180 mM NaCl (A) and non-adapted (NA) cells exposed to 0 or 180 mM NaCl. The data represent the average of 3 independent experiments. Error bars represent standard deviation. Statistical analysis was carried out by ANOVA and a T Test. N.D.: not detected.

Regarding osmoregulation, it was unexpected that 3 genes involved in the biosynthesis of (hydroxy)ectoine-diaminobutyrate-2-oxoglutarate transaminase (*ectB*, -6.5-fold), L-ectoine synthase (*ectC*, -5.6-fold) and ectoine hydroxylase (*ectD*, -8.0-fold)—were all significantly down-regulated. Since hydroxyectoine plays an important role in protecting the cells of *L*. *ferriphilum* against saline stress [4], we were interested in evaluating the intracellular content of ectoine and hydroxyectoine in adapted cells exposed to 180 mM NaCl. As shown in **Fig 3**, ectoine was not detected in either adapted or non-adapted cells. However, hydroxyectoine reached 20 nmol mg^-1^ of wet biomass (p<0.01) in non-adapted cells cultured without NaCl while it was not detected in extracts of *L*. *ferriphilum* adapted to 180 mM NaCl. Taken together, these results reinforce the idea that the 180 mM NaCl-adapted culture of *L*. *ferriphilum* does not develop an active response to osmotic stress based on the synthesis of compatible solutes. Interestingly, in non-adapted cells, the compatible solute-mediated response appears to be functionating, and in this way these cells could be actively responding to the osmotic challenge.

#### Stress response

Presumed stress response genes that exhibited a significant increase in their transcript levels encoded the following proteins: a flavohemoprotein (14.3-fold), an enzyme able to reduce nitric oxide (NO) from reactive nitrogen species (RNS) [56]; a cytochrome *c* peroxidase (10.9-fold) able to reduce periplasmic hydrogen peroxide [57]; an FAD-dependent pyridine nucleotide-disulfide oxidoreductase (5.6-fold) which catalyzes disulfide bond formation and reduction [58,59]; and a radical S-adenosyl-methionine (SAM, 5.2-fold) precursor for the biosynthesis of the antioxidant cobalamin [23]. These data strongly suggest that in *L*. *ferriphilum*, antioxidant proteins form part of the mechanisms that are activated to enable this species to face the stress induced by NaCl, and thereby manage redox homeostasis under these conditions.

**Fig 4:**
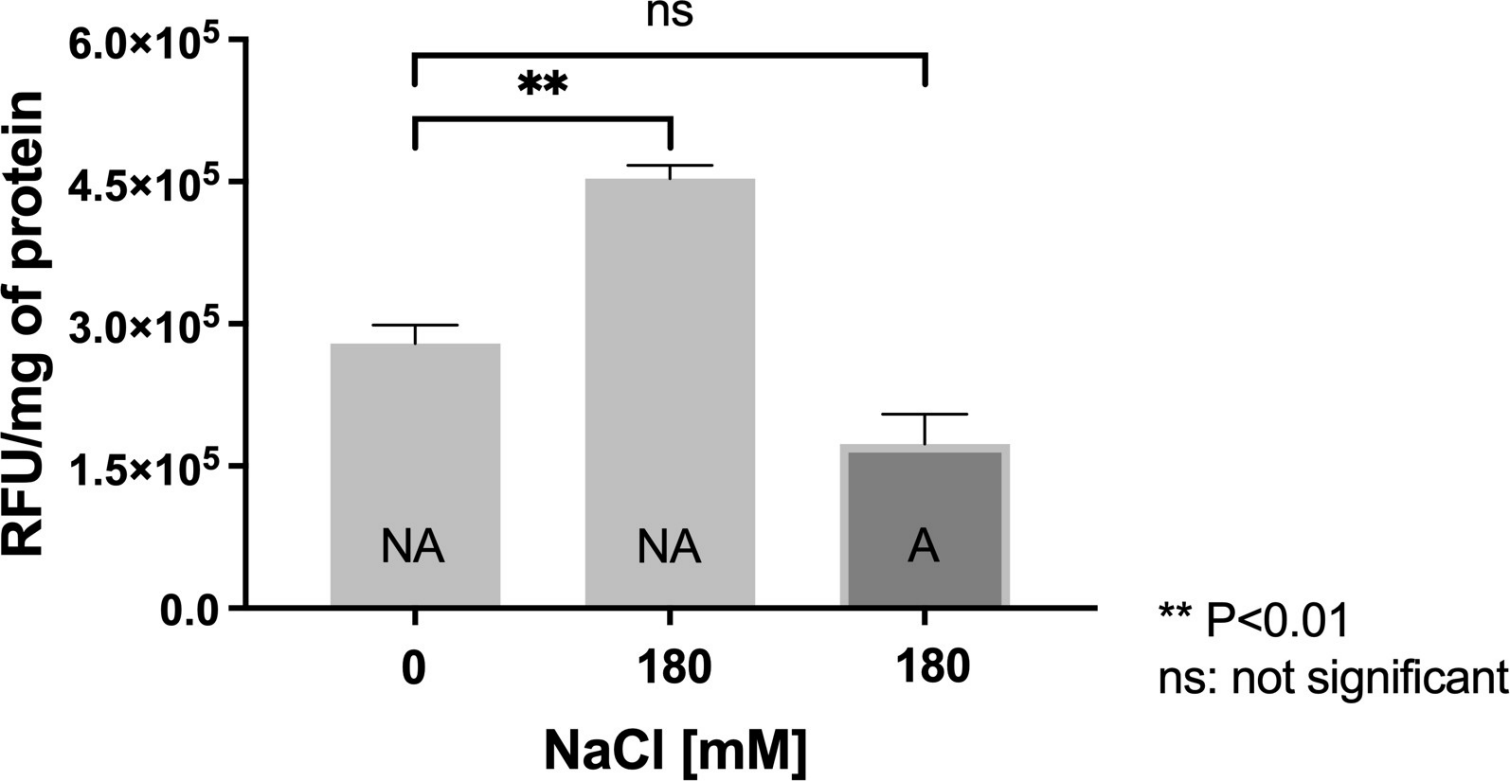
Effect of NaCl on ROS generation in *L*. *ferriphilum*. ROS were measured in adapted cells exposed to 180 mM NaCl (A) and non-adapted (NA) cells exposed to 0 or 180 mM NaCl. Cytoplasmic ROS content is expressed as relative fluorescence units (RFU) of the activated fluorescent probe H_2_DCFDA per mg of protein. The data represent the average of 3 independent experiments. Error bars represent standard deviation. Statistical analysis was carried out by ANOVA and a T Test.

In agreement with the induction of antioxidative proteins, in a previous study carried out by our research group, it was established that exposure to NaCl induced a severe condition of oxidative stress in *L*. *ferriphilum*, leading to an increase in intracellular ROS levels and activation of the antioxidant response [4]. In order to establish whether the adapted cells are able to maintain the redox balance, the intracellular ROS level was measured using a fluorescent probe as described in Material and Methods. The measurements were performed in non-adapted and adapted cultures grown in DSMZ 882 medium supplemented (or not) with 180 mM NaCl. As shown in **Fig 4**, non-adapted cells exposed to 180 mM NaCl had significantly higher intracellular ROS levels (p<0.01) compared with the control condition (without NaCl). Interestingly, salt-adapted *L*. *ferriphilum* treated with 180 mM NaCl showed similar, and even slightly lower, intracellular ROS levels compared to the control without NaCl, suggesting that these cells maintain correct redox homeostasis. This condition is most likely managed through the up-regulation of the antioxidant mechanisms described above.

Another gene that showed up-regulation (7.2-fold) encodes the IscR regulator, potentially involved in regulating the biosynthesis of [Fe-S] clusters of proteins [60]. The [Fe-S] clusters are susceptible to being oxidized by superoxide anions, thus releasing Fe^2+^, and thereby triggering Fenton chemistry and the generation of highly harmful hydroxyl radicals [16,23]. Therefore, these results imply that under high salt conditions, [Fe-S] clusters of proteins suffer oxidative damage and, in consequence, the cells respond through the activation of the biosynthesis pathway for [Fe-S] clusters.

Interestingly, the *merA* gene that encodes a mercuric reductase was over-expressed (4.3-fold) under the high-NaCl regimen. In bacteria, the mercury-resistance (*mer*) genes are activated and repressed by the metalloregulatory MerR protein, which has a high degree of selectivity for mercury (Hg^2+^) but can additionally be partially stimulated by a variety of transition metals such as Cd^2+^, Zn^2+^, Ag^+^, Au^+^, and Au^3+^ [61]. For example, in the metal-tolerant bacterium *Cupriavidus metalliduran*s, the genes *merA*, *merT*, and *merP* were up-regulated when this bacterium was exposed to cadmium [62,63]. A similar phenomenon has been described in *Nitrosomonas europaea*, since the *mer* operon was also induced by cadmium [64]. Although *merR* was not up-regulated in our study, this gene was detected in the genome of *L*. *ferriphilum* and could contribute to regulate the transcriptional activity of the *merA* gene in response to mercury or other metals. We speculate that in *L*. *ferriphilum*, chloride stress could cause oxidation of metalloproteins releasing oxidized metals to the intracellular space that may activate *merA* transcription. In *L*. *ferriphilum* this response could be relevant, since it has a high content of cytochromes and [Fe-S] proteins [32,65] that could contribute to increasing the intracellular free iron and copper contents under stress conditions. Whether the *mer* operon has a role in protection and /or avoiding toxicity toward these metals should be elucidated.

Interestingly, some genes related to stress responses were repressed. Such is the case for the gene coding for the cobalt-zinc-cadmium resistance protein CzcA (-6.8-fold), one of the three components of the CzcABC efflux pump [66]. This pump functions as a cation-proton antiporter mediating resistance against divalent metals such as cadmium (Cd^2+^), zinc (Zn^2+^), and cobalt (Co^2+^), among others [67]. As chloride exposure is known to induce cytoplasmic acidification by favoring entry of protons into the cell, the response to this anion should involve strategies that contribute to keeping the intracellular pH closer to neutrality. Thus, repression of the *czcA* gene and eventual down-regulation of CzcABC pump activity in adapted *L*. *ferriphilum* could participate towards avoiding the entry of protons into the cytoplasm.

#### Signal transduction

Among the genes that were up-regulated in the culture adapted to NaCl, we detected one gene encoding a diffusible signal factor (DSF) synthase (10.7-fold) and 3 genes coding for diguanylate cyclase phosphodiesterase (5.2, 6.6 and 11.2-fold). The protein DSF synthase (RpfF) synthesizes diffusible signal factors, widely conserved quorum sensing signals in many Gram-negative bacterial species that play important roles in regulating various biological functions such as biofilm formation, virulence, and antibiotic and stress resistance [68,69]. RpfF synthesizes DSF by dehydration of a 3-hydroxyacyl-acyl carrier protein (ACP) fatty acid intermediate and also cleaves the thioester bond linking DSF to ACP [70]. When DSFs reach a threshold concentration outside the cell, bacteria activate their cognate receptor RpfC, a hybrid membrane sensor kinase that phosphorylates the intracellular response regulator RpfG [70]. The activated RpfG possesses c-di-GMP phosphodiesterase activity, which hydrolyzes c-di-GMP to produce GMP. The change in c-di-GMP level affects the transcriptional expression of target genes, thus configuring a physiological response or modulating a biological process [70]. Therefore, based on the up-regulation of genes encoding DSF synthase and diguanylate cyclase phosphodiesterase, it is possible to infer that adaptation of *L*. *ferriphilum* to NaCl involves the activation of a cellular response mediated by DSF signals and the second messenger c-di-GMP. However, the target genes that are modulated by this mechanism remain to be elucidated.

#### Others

Other genes up-regulated by NaCl adaptation were two methyl-accepting chemotaxis proteins (5.4-fold and 5.1-fold) and a flagellin protein FlaB (4.4-fold) which are related with movement of microorganisms in response to chemical gradients, and biosynthesis of flagella, respectively [71]. An effect of osmolarity challenges on flagellar function has previously been reported in bacteria. Specifically, in *Desulfovibrio vulgaris*, cells were observed to be highly motile when subjected to salt stress and several key chemotaxis genes were very highly and reproducibly up-regulated [72]. More recently, *Escherichia albertii* showed swimming motility when cultured at low osmotic pressure. Under this condition, the biosynthesis of flagella was also induced [73]. It has been predicted that flagellar induction increases *E*. *albertii* survival in intestinal epithelial cell cultures. Whether motility and flagellum assembly are activated by NaCl exposure, and the corresponding impact of their activation on adaptation and fitness of leptospirilli should be addressed.

Another group of genes overexpressed in the NaCl-adapted culture encodes a transposase (7.7-fold), a phage-related integrase (5.4-fold), a DNA-binding protein HU (4.3-fold) and a shufflon-specific DNA recombinase (4.2-fold). All are involved in bacterial DNA transaction systems including transposition and recombination, among others [74]. Therefore, genetic/genomic modifications could underlie physiological stress responses and/or may pre-adapt a small subset of the population to face this environmental stress.

## Conclusions

Despite its high chloride sensitivity, *L*. *ferriphilum* could be stably adapted to 180 mM NaCl. In adapted cells, the MIC and thus tolerance to NaCl increased considerably compared to non-adapted non exposed cells. The MIC of adapted and non-adapted cells was shown to be directly dependent on the pH of the medium, and so the comparison of tolerance to chloride or other anions in acidophilic microorganisms should be carried out whilst strictly monitoring the pH of the growth medium.

Transcriptomic data and experimental validations showed that the most significant responses of *L*. *ferriphilum* to chloride adaptation included neutralization and/or expulsion of protons through activation of carbonic anhydrase, respiratory cytochrome *c* oxidase and sulfide:quinone reductase. Thus, the regulation of pH homeostasis seems to play a key role in the adaptive response. Towards the same goal, a cation/proton antiporter system CzcA that extrudes cations through the entry of protons was down-regulated. In addition, the increase in respiratory activity and oxygen consumption correlated with activation of antioxidant responses in which genes encoding for ROS scavenging properties and biomolecule protection seem to play a relevant role in controlling the intracellular ROS level and the redox status of adapted cells. The response detected shows that oxidative stress is an important element of the toxicity induced by chloride, and this could largely explain the reason why iron-oxidizing microorganisms have been reported to be more sensitive to the presence of anions than sulfur-oxidizers or other acidophiles [75]. Under cultivation conditions, iron-oxidizing microorganisms are exposed to high concentrations of iron as an energy substrate, while sulfur oxidizers are exposed to trace concentrations of this element that is used only as a micronutrient. Since ferrous iron can trigger Fenton chemistry, its presence in high concentrations leads to a higher risk of redox stress, making the microorganisms more sensitive to other oxidative stress elicitors. Chloride adaptation also correlated with a predicted increase in chemotaxis and biosynthesis of flagella, and predicted cellular communication and signaling via DSFs and c-di-GMP. Finally, an induction of genetic/genomic modifications by transposition and/or recombination also seemed to form part of the adaptive response to NaCl exposure. Although there was an increase in the activity of the electron-transport chain that likely led to an increase in ATP and NAD(P)H synthesis, carbohydrate metabolism and synthesis of polysaccharide polymers of the cell surface seemed to suffer significant decreases. Surprisingly, the canonical osmotic stress response did not appear to be necessary in salt-adapted cells, since genes for biosynthesis of the compatible solutes ectoine and hydroxyectoine were down-regulated, and only hydroxyectoine could be detected and only in non-adapted cells without NaCl. Our results suggest that *L*. *ferriphilum* might have a response to long-term NaCl exposure that is different from other bacteria since it does not involve the upregulation of canonical mechanisms for facing osmotic stress. This study thus provides an important reference for future studies on NaCl adaptation in acidophilic bacteria.

## Supporting information

S1 Table Up-regulated hypothetical genes in *L*. *ferriphilum* NaCl-adapted cells. (XLSX)

S2 Table Down-regulated hypothetical genes in *L*. *ferriphilum* NaCl-adapted cells. (XLSX)
